# Effect of autonomic nervous system resection extent on urinary dysfunction in robotic rectal cancer surgery

**DOI:** 10.1002/ags3.12878

**Published:** 2024-11-04

**Authors:** Sodai Arai, Hiroyasu Kagawa, Akio Shiomi, Yusuke Yamaoka, Shoichi Manabe, Chikara Maeda, Yusuke Tanaka, Shunsuke Kasai, Akifumi Notsu, Yusuke Kinugasa

**Affiliations:** ^1^ Division of Colon and Rectal Surgery Shizuoka Cancer Center Shizuoka Japan; ^2^ Department of Gastrointestinal Surgery Institute of Science Tokyo Tokyo Japan; ^3^ Clinical Research Promotion Unit Shizuoka Cancer Center Shizuoka Japan

**Keywords:** autonomic nervous system, pelvic plexus, rectal cancer, robotic surgery, urinary dysfunction

## Abstract

**Aim:**

We investigated whether autonomic nervous system resection during robotic rectal surgery contributes to urinary dysfunction and to what extent.

**Methods:**

This retrospective cohort study included patients who underwent rectal surgery for primary rectal cancer between December 2011 and April 2021. We identified urinary dysfunction risk factors and examined the effect of autonomic nervous system resection extent on urinary dysfunction occurrence, with urinary dysfunction defined as a residual urine volume of >50 mL. Urinary dysfunction with no improvement over 1 y was defined as permanent urinary dysfunction.

**Results:**

Of 1017 eligible patients, 78 (7.7%) required autonomic nervous system resection. Lateral lymph node dissection was performed in 357 patients (35.1%). Urinary dysfunction was observed in 102 patients (10.0%). We studied 32 (41.0%) of 78 patients who underwent autonomic nervous system resection and 82 (23.0%) of 357 patients who underwent lateral lymph node dissection presented with urinary dysfunction. Multivariate analysis revealed that lateral lymph node dissection and autonomic nervous system resection were significant predictors of urinary dysfunction. The urinary dysfunction incidence was notably higher in patients with autonomic nervous system unilateral total resection of at least one side than in those with bilateral preservation (65.4% vs. 28.8%, *p* < 0.01), and permanent urinary dysfunction exclusively occurred in these patients.

**Conclusion:**

In robotic surgery, autonomic nervous system resection and lateral lymph node dissection were independent risk factors for urinary dysfunction. Furthermore, the extent of autonomic nervous system resection may increase the risk of permanent urinary dysfunction.

## Graphical abstract

In rectal cancer surgery, this study examined the risk factors for urinary dysfunction and the effect of autonomic nervous system resection extent on urinary dysfunction. Autonomic nervous system resection and lateral lymph node dissection were independent risk factors for urinary dysfunction. The extent of autonomic nervous system resection was associated with increased urinary dysfunction.

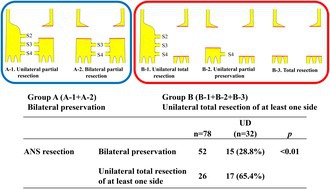

## 1 INTRODUCTION

Robotic surgery could potentially surpass open or laparoscopic surgery in technological sophistication, primarily due to its exceptional three‐dimensional visualization, magnification capabilities, and improved maneuverability enabled by multijoint forceps. In Japan, both mortality and open conversion rates for robotic rectal surgery were 0% in the Japanese National Clinical Database, a nationwide web‐based data entry system, suggesting the safety and usefulness of robotic rectal surgery.^1^ The REAL trial, a randomized controlled trial (RCT) for rectal cancer, reported that robotic surgery was effective compared to laparoscopic surgery regarding blood loss, open conversion rate, circumferential resection margin, postoperative complication rate, and postoperative hospital stay, although operating time was not different.^2^

Postoperative urinary dysfunction (UD), particularly urinary retention, is a major complication that requires preventive measures for rectal cancer, owing to its effects on patients' quality of life. Many previous reports of rectal surgery have shown a high incidence of UD, ranging from 2.7% to 48%.^3^, ^4^, ^5^, ^6^, ^7^, ^8^, ^9^, ^10^, ^11^, ^12^, ^13^, ^14^ Several reports showed that robotic surgery reduced UD compared to open or laparoscopic surgery, but the risk factors for UD in robotic surgery are unclear.^3^, ^5^ The autonomic nervous system (ANS), especially S2–S4 pelvic nerves and pelvic plexus, is involved in urinary function,^15^ and some reports suggest ANS resection is a risk factor for UD.^13^, ^16^ The Japanese Society for Cancer of the Colon and Rectum (JSCCR) guidelines state that urinary function can be maintained to a certain degree if one side of the pelvic plexus is preserved.^15^ Studies have indicated that, in open surgery, urinary function can be improved if S4 is preserved^17^ and that preserving one side of the pelvic nerve plexus can help maintain some aspect of the urinary function^15^, ^16^; however, the relationship between the extent of ANS resection and the incidence or improvement of UD remains unclear.

The characteristics of robotic surgery enable better recognition of the ANS and the prehypogastric nerve fascia that covers it, particularly in the narrow or deep pelvis.^5^, ^18^, ^19^ Therefore, the relationship between the extent of ANS resection and UD during robotic surgery should be examined in more detail. In this study, we aimed to confirm whether ANS resection is a risk factor for UD after robotic rectal surgery and evaluate the impact of the extent of ANS resection on the incidence of UD.

## 2 METHODS

### 2.1 Study design and patients

We retrospectively examined all patients who underwent robotic surgery for primary rectal adenocarcinoma cancer at Shizuoka Cancer Center in Japan between December 2011 and April 2021. Patient data were collected, including perioperative characteristics, postoperative complications, and pathological characteristics. Patients who relapsed and underwent preoperative urostomy or total pelvic exenteration, and those with preoperative UD were excluded because of difficulties in evaluating UD. In rectal surgery, high anterior resection is a procedure that requires little or no mobilization below the peritoneal reflection, so there is little possibility of injury to the ANS. Hence, patients who underwent high anterior resection were excluded.

Preoperative evaluation included endoscopic biopsy for histological confirmation of adenocarcinoma, computed tomography, magnetic resonance imaging, and barium enema for TNM classification.^20^ Preoperative chemoradiation therapy (CRT) was administered to patients predicted to have positive circumferential resection margins in surgery without CRT, individuals for whom anal preservation was feasible with a tumor reduction effect, or patients for whom anticipated urinary tract changes could be mitigated. CRT, involving a total radiation dose of 50.4 Gy in 28 fractions, was administered with systemic capecitabine chemotherapy for 5–6 weeks. This study was designed in accordance with the Strengthening the Reporting of Observational Studies in Epidemiology (STROBE) statement. Consent in this study was obtained from all patients. The Institutional Review Board of Shizuoka Cancer Center approved all study protocols (institutional code: J2021‐77‐2021‐1‐3). The study conforms to the provisions of the Declaration of Helsinki (as revised in Fortaleza, Brazil, October 2013).

### 2.2 Surgical procedures

We performed conventional total mesorectal excision (TME) or tumor‐specific mesorectal excision (TSME), as indicated in a previous report.^21^ The ANS was preserved in cases where preservation was possible, but in cases where the tumor had invaded beyond the mesorectum, combined resection of adjacent organs and/or ANS resection was performed as necessary to achieve a complete circumferential resection margin. The lateral lymph node dissection (LLD) criteria were based on the JSCCR guidelines. They were performed when the lower border of the tumor was located distal to the peritoneal reflection (lower rectum), and the tumor depth was cT1/cT2 with preoperative lateral lymph node metastasis or cT3/cT4.^15^ LLD was performed bilaterally. However, in patients with severe comorbidities, such as uncontrolled diabetes, or those over 75 y, only unilateral LLD was performed, or LLD was omitted altogether.

### 2.3 Definition of postoperative UD

UD was defined as a residual urine volume >50 mL based on previous studies.^5^, ^14^ Residual urine volume was measured using the Bladder Scan System BVI6100 (Sysmex, Kobe, Japan), which uses ultrasound. Residual urine volume was measured one or 2 d before surgery and after removing the urethral catheter on the fifth postoperative day; the evaluation was performed at least twice. Patients who were administered medication for UD preoperatively or had preoperative residual urine ≥50 mL were excluded from the analysis of UD. Residual urine volume was calculated once when the sum of discharged and residual urinary volume was >150 mL.^14^ The grade of UD was classified according to the Clavien–Dindo (CD) classification.^22^ Patients were followed‐up every 3 or 6 mo for 5 y after surgery. Improvement in UD was defined as controlled voiding without self‐catheterization. Permanent UD was defined as UD that persisted for more than 12 mo postoperatively and required ongoing catheterization.

### 2.4 ANS resection pattern

Regarding ANS preservation, the Japanese Classification of Colorectal, Appendiceal, and Anal Carcinoma classifies preservation of the autonomic nerves as AN1‐4 (Figure S1)^20^; however, this classification is not used for UD evaluation. Hypogastric nerves control ejaculation, and according to our research no studies have assessed the relationship between the hypogastric nerves and urinary function. In this study, ANS resection meant that one of the pelvic nerves/plexuses from S2 to S4 was resected, and the pelvic nerves/plexus from S2 to S4 were treated as a narrowly defined ANS. We retrospectively reviewed surgical records and collected data on the extent of pelvic nerves/plexus resection. We further analyzed the relationship between the extent of resection of the pelvic nerves/plexuses from S2 to S4, which is closely related to urinary function and UD. The ANS resection was divided into the following five groups according to the extent of ANS resection—unilateral partial resection (A‐1), bilateral partial resection (A‐2), unilateral total resection (B‐1), unilateral partial preservation (B‐2), and total resection (B‐3)—and the incidence of UD was compared.

### 2.5 Surgical outcomes

The surgical outcomes were assessed, including the incidence of UD and other postoperative complications within 30 d after surgery. Postoperative complication grades were evaluated based on the CD classification.^22^ The extent of ANS resection was dichotomized into two major categories: Group A (bilateral preservation, A‐1 and A‐2) and Group B (unilateral total resection of at least one side, B‐1, B‐2, and B‐3), and the incidence of UD was evaluated.

### 2.6 Statistical analysis

Categorical variables were presented as numbers (patient percentages) and analyzed using Fisher's exact test. Continuous variables are presented as medians (range) and were analyzed using the Mann–Whitney *U* test. Risk factors for postoperative UD were identified using univariate and multivariate logistic regression analyses. We used a Directed Acyclic Graph (DAG) for choosing the factors in the multivariate analysis (Figure S2). DAGs are used to estimate a causal effect of a factor on an outcome.^23^ Hence, our findings about the effects of factors from the multivariate analysis were causation and not association. However, in accordance with the tradition of this research field, factors having a causal effect were referred to as independent risk factors or predictors in this article. The intermediate variable, blood loss, was excluded from the multivariate analysis. All *p*‐values were two‐sided, and differences were considered statistically significant at *p* < 0.05. Statistical analysis was performed using EZR (v. 1.54, Saitama Medical Center, Jichi Medical University, Japan), a graphical user interface for R (The R Foundation for Statistical Computing, Vienna, Austria).

## 3 RESULTS

### 3.1 Patient characteristics

In total, 1017 patients were included in this study (Figure S3). Patient characteristics are shown in Table 1. The median age of the patients was 66 y (28–93 y), and 678 (66.7%) were male. More than half of the patients had tumors in the lower rectum (58.6%); 67.1% were categorized as clinical T stages 3 or 4, with 5.8% undergoing neoadjuvant CRT.

**TABLE 1:** Perioperative patient characteristics.

Variables	*n* = 1017
Age, y	66 (28–93)
Sex	
Male	678 (66.7)
Female	339 (33.3)
BMI, kg/ m^2^	23.0 (14.3–47.9)
ASA score	
I	231 (22.7)
II	733 (72.1)
III	53 (5.2)
Diabetes mellitus	186 (18.3)
Hypertension	363 (35.7)
Tumor location^a^	
Upper or middle rectum	421 (41.4)
Lower rectum	596 (58.6)
c/yc T stage	
X/ is/ 1	217 (21.3)
2	118 (11.6)
3	500 (49.2)
4	182 (17.9)
c/yc N stage	
0	453 (44.5)
1	295 (29.0)
2	269 (26.5)
c/yc Stage	
0	2 (0.2)
I	283 (27.8)
II	161 (15.8)
III	489 (48.1)
IV	82 (8.1)
Neoadjuvant chemoradiation therapy	59 (5.8)
Procedure	
Low anterior resection	768 (75.5)
Intersphincteric resection	124 (12.2)
Abdominoperineal resection	115 (11.3)
Other	10 (1.0)
Operative time, min	270 (109–730)
Blood loss, mL	10 (0–2230)
Lateral lymph node dissection	357 (35.1)
Combined resection of adjacent organs	66 (6.5)
Autonomic nervous system resection	78 (7.7)
Conversion to open surgery	1 (0.1)
Tumor size, cm	4.0 (0.5–15.0)
Histologic type	
Tub/ Pap	983 (96.7)
Por/ Muc/ Sig	34 (3.3)

*Note*: Unless indicated otherwise, values are presented as the median (range) or numbers (percentages).

Abbreviations: ASA, American Society of Anesthesiologists; BMI, body mass index; Pap, papillary adenocarcinoma; Muc, mucinous adenocarcinoma; Por, poorly differentiated adenocarcinoma; Sig, signet‐ring cell carcinoma; Tub, tubular adenocarcinoma.

^a^ Upper or middle rectum was defined as the lower border of the tumor located proximal to the peritoneal reflection, and lower rectum as distal to the peritoneal reflection.

Of the included patients, 939 (92.3%) underwent ANS‐preserving surgery. However, 78 (7.7%) required ANS resection. LLD was performed in 35.1% of the patients. A total of 6.5% of the patients underwent combined resection of the adjacent organs. The median operative time was 270 min (109–730 min), and the median blood loss was 10 mL (0–2230 mL). Only one patient required conversion to open surgery.

### 3.2 Postoperative outcomes

The postoperative complication outcomes are shown in Table 2. UD was observed in 102 patients (10.0%). UD was found in only 12 (2.0%) of the 602 TME/TSME cases with complete ANS preservation (without LLD and combined resection of adjacent organs). The incidence of Grade II and III postoperative complications was 22.6% and 5.8%, respectively. Anastomotic leakage was observed in 35 patients (3.9%) of the patients with anastomosis. The median postoperative hospital stay was 7 d (6–47 d).

**TABLE 2:** Postoperative outcomes.

Variables	*n* = 1017
Urinary dysfunction	
All case	102 (10.0)
TME/ TSME with ANS preservation^a^	12 (2.0)
Residual urine volume, mL	
0–50	915 (90.0)
50–100	8 (0.8)
100–150	14 (1.4)
150–200	23 (2.2)
200<	57 (5.6)
CD ≥ Grade III	59 (5.8)
CD ≥ Grade II	230 (22.6)
SSI	
All	75 (7.4)
Superficial	12 (1.2)
Deep	6 (0.6)
Organ space	59 (5.8)
Anastomotic leakage^b^	35 (3.9)
Ileus	44 (4.3)
Urinary infection	27 (2.7)
Pneumonia	15 (1.5)
Intraabdominal bleeding	12 (1.2)
Anastomotic bleeding	11 (1.1)
Delirium	9 (0.9)
Catheter‐related infection	7 (0.7)
Obturator nerve dysfunction	3 (0.3)
Others	51 (5.0)
Postoperative hospital stay, day	7 (6–47)

Note: Unless indicated otherwise, values are presented as the median (range) or numbers (percentages).

Abbreviations: CD, Clavien–Dindo, SSI, surgical site infection; TME, total mesorectal excision, TSME, tumor‐specific mesorectal excision.

^a^ The number of TME/ TSME with ANS preservation was 602.

^b^ Data were analyzed in patients with anastomosis.

### 3.3 Risk factors for postoperative UD

In the univariate analysis, several factors were significantly associated with UD (Table 3). From a total of 16 (27.1%) of the 59 patients who underwent CRT, 19 (28.8%) of the 66 patients who underwent adjacent organs resection, 32 (41.0%) of the 78 patients who underwent ANS resection, and 82 (23.0%) of the 357 patients who underwent LLD had UD. Other risk factors for UD identified in the univariate analysis included tumor in the lower rectum, cT3/T4, abdominoperineal resection, blood loss ≥50 mL, and tumor size ≥6 cm. Multivariate analysis was performed using factors selected according to the DAG, except the intermediate variable, blood loss. Multivariate analysis showed that LLD (odds ratio [OR]: 6.18; 95% confidence interval [CI]: 3.26–11.7, *p* < 0.01) and ANS resection (OR: 4.50; 95% CI: 2.51–8.09, *p* < 0.01) were significant predictors of UD.

**TABLE 3:** Univariate and multivariate analyses of UD.

Variables		Univariate		Multivariate		
	*n*	UD	*p* value	OR	95% C.I.	*p* value
Sex						
Female	339	32 (9.4)	0.74			
Male	678	70 (10.3)				
Age, y						
< 65	452	48 (10.6)	0.60			
≥ 65	565	54 (9.6)				
Diabetes mellitus						
No	831	79 (9.5)	0.28			
Yes	186	23 (12.4)				
Neoadjuvant chemoradiation therapy						
No	958	86 (9.0)	<0.01			
Yes	59	16 (27.1)				
Tumor location						
Upper/middle	421	24 (5.7)	<0.01			
Lower	596	78 (13.1)				
c/yc T stage						
TX/Tis/T1/T2	335	10 (3.0)	<0.01			
T3/ T4	682	92 (13.5)				
Procedure						
LAR/ ISR	902	77 (8.5)	<0.01			
APR	115	25 (21.7)				
Lateral lymph node dissection						
No	660	20 (3.0)	<0.01	6.180	3.260–11.70	<0.01
Yes	357	82 (23.0)				
Autonomic nervous system resection						
No	939	70 (7.5)	<0.01	4.500	2.510–8.090	<0.01
Yes	78	32 (41.0)				
Adjacent organs resection						
No	951	83 (8.7)	<0.01			
Yes	66	19 (28.8)				
Blood loss, mL						
< 50	860	71 (8.3)	<0.01			
≥ 50	157	31 (19.7)				
Tumor size, cm						
<6	781	58 (7.4)	<0.01			
≥6	236	44 (18.6)				
Histologic type						
Tub/ Pap	983	96 (9.8)	0.14			
Por/ Muc/ Sig	34	6 (17.6)				

*Note*: Values in parentheses represent percentages. Multivariate analysis was performed with all factors in univariate analysis except the intermediate variable, blood loss, and only exposure variables of interest are shown.

Abbreviations: APR, abdominoperineal resection; C.I, confidence interval; HAR, high anterior resection; ISR, intersphincteric resection; LAR, low anterior resection; Muc, mucinous adenocarcinoma; OR, odds ratio; Pap, papillary adenocarcinoma; Por, poorly differentiated adenocarcinoma; Sig, signet‐ring cell carcinoma; Tub, tubular adenocarcinoma; UD, urinary dysfunction.

### 3.4 Relationship between ANS resection extent and UD

**FIGURE 1:**
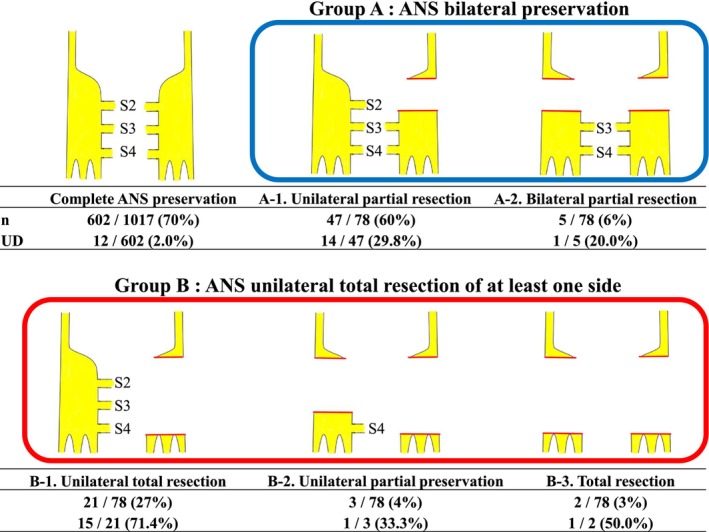
Incidence of UD by the extent of ANS resection. The complete ANS preservation group comprised TME/TSME cases with complete ANS preservation (without LLD and combined resection of adjacent organs).

**FIGURE 2:**
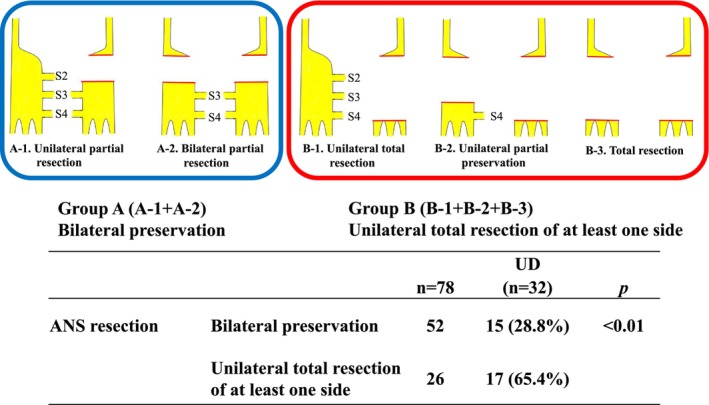
Risk factors for UD due to ANS resection extent group.

Eighty patients underwent ANS resection (Figure 1). In Group A (bilateral preservation group), UD was observed in 14 of 47 patients (29.8%) in the A‐1 group, which was the most minor extent of resection. In Group B (unilateral total resection of at least one side), UD was observed in one of the two patients (50.0%) in the B‐3 group, which was the greatest extent of resection. UD was most frequent in the B‐1 group, occurring in 15 (71.4%) of the 21 patients. Comparing Groups A (ANS bilateral preservation group; A‐1 and A‐2) and B (ANS unilateral total resection of at least one side; B‐1, B‐2, and B‐3), the incidence of UD was 28.8% and 65.4%, respectively (*p* < 0.01), revealing a higher incidence of UD in Group B (Figure 2).

In the 30 patients who underwent ANS resection with UD, 86% of patients in Group A (A‐1 and A‐2) showed withdrawal from self‐catheterization within 3 mo after surgery, and all patients showed withdrawal from self‐catheterization within 1 y. For Group B (B‐1, B‐2, and B‐3), 75% of the patients showed withdrawal from self‐catheterization within 3 mo after surgery, but two patients (12%) had permanent UD (Table 4). No significant difference in withdrawal from self‐catheterization was observed between the two groups.

**TABLE 4:** ANS resection extent group in relation to withdrawal from self‐catheterization and permanent UD.

UD with ANS resection (*n* = 30^a^)		Group A bilateral preservation (A‐1 + A‐2)	Group B unilateral total resection of at least one side (B‐1 + B‐2 + B‐3)	
	Months	*n* = 14	*n* = 16	*p* value
Withdrawal from self‐catheterization	≤ 1	6 (43%)	9 (56%)	0.72
	≤ **3**	12 (86%)	12 (75%)	0.66
	≤ 6	13 (93%)	13 (81%)	0.60
	< 12	14 (100%)	14 (88%)	0.49
Permanent UD		0	2 (13%)	0.49

Abbreviations: ANS, autonomic nervous system resection; UD, urinary dysfunction.

^a^ Two cases could not be evaluated due to follow‐up difficulties.

## 4 DISCUSSION

This study examined the relationship between the extent of ANS resection and UD, involving a large number of cases in robotic surgery, offering potential benefits regarding ANS preservation. Our research showed that robotic rectal surgery exhibited a UD rate of 10.0%, although notably lower in cases of TME/TSME (only 2%). Furthermore, our findings established ANS resection and LLD as independent risk factors for UD. In robotic surgery, the magnification effect and multijoint functionality effectively expand the surgical field, enabling surgeons to recognize the dissection layer, thereby facilitating ANS preservation. Notably, the extent of ANS resection was shown to impact the incidence of UD, with ANS unilateral total resection of at least one side emerging as a risk factor for permanent UD. The incidence of UD was lower in the ANS partial preservation group (Group B‐2) and ANS total resection group (Group B‐3) than in the ANS unilateral total resection group (Group B‐1), indicating that ANS resection may have been contemplated but not performed.

The UD evaluation method in this study was based on the JCOG0212,^14^ the largest RCT evaluating UD, which was 46% in the TME/TSME group and 48% in the TME/TSME and LLD groups. Notably, the JCOG0212 was primarily designed for open surgery, employing an approach different from that used in our present study. The robotic magnification effect has made recognizing and preserving the ANS, the prehypogastric nerve fascia, and the Denonvilliers' fascia possible. The Denonvilliers' fascia has been reported to affect UD when resected,^24^, ^25^ but until the 2000s, the Denonvilliers' fascia was often resected in rectal surgery.^26^ At our institution, the Denonvilliers' fascia, which would affect UD if resected, is preserved in rectal surgery. Therefore, the incidence of UD was lower in this study than in the JCOG0212.

Using multivariate analysis, we identified LLD as an independent risk factor for UD in addition to ANS resection. Although it has been reported that LLD is a risk factor for UD,^27^ JCOG0212 reported that the incidence of UD was comparable between TME and TME with LLD^14^ and that LLD with ANS preservation was not a risk factor for UD.^9^, ^13^ Another study reported that LLD can cause UD due to thermal damage to ANS and vessels. The incidence of UD is increased by resection of the inferior vesical artery,^28^ suggesting that potential thermal damage to ANS and the resection of the vesical arteries, including the inferior vesical artery, may affect LLD.

Previous studies have demonstrated a wide range of UD incidences due to the heterogeneity in evaluating UD (Table 5).^3^, ^4^, ^5^, ^6^, ^7^, ^8^, ^9^, ^10^, ^11^, ^12^, ^13^, ^14^ Studies that defined UD using residual urine volume also reported different incidences (range: 10%–48%). However, when limited to minimally invasive surgery, the range was 10.0%–29.1%.^5^, ^10^, ^11^, ^12^, ^13^, ^14^ Kim et al reported that robotic surgery may reduce the incidence of UD, as determined by the presence or absence of urinary retention.^3^ However, studies on the quantitative evaluation of UD using robotic surgery are lacking in the literature. In our study, defining UD as a residual urine volume ≥ 50 mL represented the smallest threshold compared to previous reports. With an overall UD incidence rate of 10.0% and a mere 2.0% among TME/TSME patients, our results align favorably with this definition. This suggests that robotic surgery is an effective approach to reducing UD.

**TABLE 5:** Review of UD.

Author	Year	Country	Design	Number (n)	Procedure (all TME)	Definition of UD	UD (%)
Hur H^6^	2013	Korea	Cohort	97	Open (42%) versus Lap (58%)	Increase in IPSS from preoperation (6 mo after surgery)	Lap 10.7% versus Open 14.6% (no SD)
Kneist W^7^	2005	Germany	Case series	210	Open	Urinary retention	3.8%
Patriti A^4^	2009	Italy	RCT	66	Ro (44%) versus Lap (56%)	Urinary retention	Ro 3.4% versus Lap 2.7% (no SD)
Kim H J^3^	2018	Korea	Case series	85	Ro( 59%) versus Lap (41%)	Urinary retention	Ro 4.0% versus Lap 20.0% (SD)
Morino M^8^	2009	Italy	Case series	50	Lap	Clavien‐Dindo Grade ≥2	14%
Toritani K^9^	2019	Japan	Case–control	887	Open (78.5%), Lap (21.5%)	Clavien–Dindo Grade ≥2	8.8%
Kim H O^10^	2016	Korea	Case series	110	Lap	Residual urine volume (≥200 mL)	29.1%
Hamamoto H^11^	2020	Japan	Case series	104	Lap	Residual urine volume (≥150 mL)	17.0%
Sterk P^12^	2005	Germany	Case series	52	Open	Residual urine volume (≥100 mL), uroflowmetry	24.4%
Shiraishi T^13^	2021	Japan	Case series	231	TaTME	Residual urine volume (≥100 mL), IPSS	12.1%
Ito M^14^	2018	Japan	RCT	701	Open	Residual urine volume (≥50 mL)	TME/TSME 46%, TME/TSME+LLD 48%
Yamaoka Y^5^	2021	Japan	Case–control	337	Ro (54%), Lap (40%), Open (6%)	Residual urine volume (≥50 mL)	10.0%
Our study	2023	Japan	Case series	1017	Ro	Residual urine volume (≥50 mL)	10.0%, TME/TSME 2.0%

*Note*: We sorted a review based on the evaluation method of UD, arranged in order of decreasing standard residual urine volume, and those with the same residual urine volume were arranged in chronological order.

Abbreviations: IPSS, International Prostatic Symptoms Score; Lap, laparoscopic surgery; RCT, randomized controlled trial; Ro, robotic surgery; SD, significant difference; TaTME, transanal total mesorectal excision; TME, total mesorectal excision; TSME, Tumor‐specific mesorectal excision; UD, urinary dysfunction.

Previous studies have reported the following risk factors for UD: older age (>65 y), male sex, tumor depth (pT3/4), tumor location in the lower rectum, abdominoperineal resection, diabetes mellitus, tumor size (≥4 cm), blood loss (≥500 mL), and LLD.^5^, ^9^, ^10^, ^11^, ^13^, ^14^, ^27^ In our study, certain factors reported as risk factors in previous research were significant in the univariate analysis but not in the multivariate analysis. Our study uniquely identified ANS resection and LLD as independent risk factors for UD. This suggests that other studies might not have included ANS resection and LLD as separate risk factors, which could explain the variation in their findings.

In the ANS, S2‐4 plays a pivotal role in urinary function, particularly in bladder contraction.^29^ It runs along the lateral pelvic wall toward the bladder and follows the arteries to the bladder as the bladder plexus. Therefore, surgery in the dissected layer beyond the TME may result in resection of the ANS, leading to UD. If the nerve is not completely transected, it can regenerate, and an improvement in UD can be expected.^12^ However, UD that persists for a year is considered permanent.^30^ A few studies assessing the relationship between ANS preservation and the incidence of UD exist, and they all reported that ANS resection increases the incidence of UD. Kneist et al found that the incidence of UD was significantly higher with ANS resection in open surgery, 1.2% with complete ANS preservation and 14.3% without complete ANS preservation.^7^ In multivariate analysis, incomplete ANS preservation was an independent risk factor for UD. Akasu et al reported a significant difference in the duration of UD improvement in open surgery with ANS preservation, unilateral preservation, and total resection between the groups, and that ANS preservation improved UD earlier.^31^ Similarly, Shiraishi et al reported that UD was significantly higher in transanal total mesorectal excision with ANS unilateral total resection of at least one side, at 83.3%, and remained as high as 50% at 6 mo postoperatively.^13^ Based on these previous reports, the relationship between the extent of ANS resection and the incidence of UD and incidence of permanent UD remains unclear; thus, our study is significant in this aspect. Another possible risk factor for permanent UD is the possibility that CRT may have had an effect, since the two patients with permanent dysfunction were both performed with CRT.

This study has some limitations. First, it was a retrospective study conducted at a single institution. Second, only a small portion of the patients underwent ANS resection (*n* = 78), with a similarly small number of patients in the partial preservation and total resection groups. Therefore, further multicenter studies with larger sample sizes are required. Since this was a single‐institution study, the objective evaluation method of UD was standardized, and only robotic surgeries in which the ANS could be recognized were included. In addition, this study focused on the pelvic nerves/plexuses related to urinary function and examined the effect of ANS resection on urinary function. Some of the possible causes of UD included the effect of thermal conduction and unexpected injury or compression by forceps without tactile sensation in robotic surgery. Energy settings may also have an effect on thermal conduction. In future robotic surgeries, it will be necessary to select the dissection layer according to the depth of the tumor, but it will be necessary to perform surgery using a dissection layer that can reliably preserve the ANS to reduce the incidence of UD. In summary, the findings of this study are useful for examining UD in patients with rectal cancer.

## 5 CONCLUSION

In robotic surgery, ANS resection and LLD were independent risk factors for UD. The extent of ANS resection affects the incidence of UD, and ANS unilateral total resection of at least one side may be a risk factor for permanent UD. UD can be improved by preserving the ANS on bilateral sides, even if only partially.

## AUTHOR CONTRIBUTIONS

Sodai Arai, Hiroyasu Kagawa, Akio Shiomi, Yusuke Yamaoka, Shoichi Manabe, Chikara Maeda, Yusuke Tanaka, Shunsuke Kasai, Akifumi Notsu, and Yusuke Kinugasa contributed to conceptualization, article writing, and editing.

## FUNDING INFORMATION

No funding was received for this research.

## CONFLICT OF INTEREST STATEMENT

Akio Shiomi received lecture fees from Intuitive Surgical. Yusuke Kinugasa is an editorial member of *Annals of Gastroenterological Surgery*, and received lecture fees from Johnson and Johnson, Intuitive Surgical, and Medtronic.

## ETHICS STATEMENT

Approval of the research protocol by an Institutional Reviewer Board: The protocol of this study was approved by the Institutional Review Board of Shizuoka Cancer Center (institutional code: J2021‐77‐2021‐1‐3). It was in accordance with the ethical standards of the responsible committee for human experimentation and with the Helsinki Declaration of 1964 and later versions.

Informed Consent: N/A.

Registry and the Registration No. of the study/trial: N/A.

Animal Studies: N/A.

## Supporting information


**Figure S1:** Preservation of autonomic nerves on Japanese Classification of Colorectal, Appendiceal, Anal Carcinoma.

**Figure S2:** Directed Acyclic Graph (DAG) on UD.

**Figure S3:** Patient flow chart.

## Data Availability

The data analyzed during the current study are available from the corresponding author upon reasonable request.
