# Supplementary material for: Effect of autonomic nervous system resection extent on urinary dysfunction in robotic rectal cancer surgery
Source: Ann Gastroenterol Surg. 2024 Nov 4;9(3):476–85. doi: 10.1002/ags3.12878 (PMC12080188; doi:10.1002/ags3.12878)
Supplement: Supplementary file 1 — Figure S1: Preservation of autonomic nerves on Japanese Classification of Colorectal, Appendiceal, Anal Carcinoma. [file AGS3-9-476-s001.pdf]

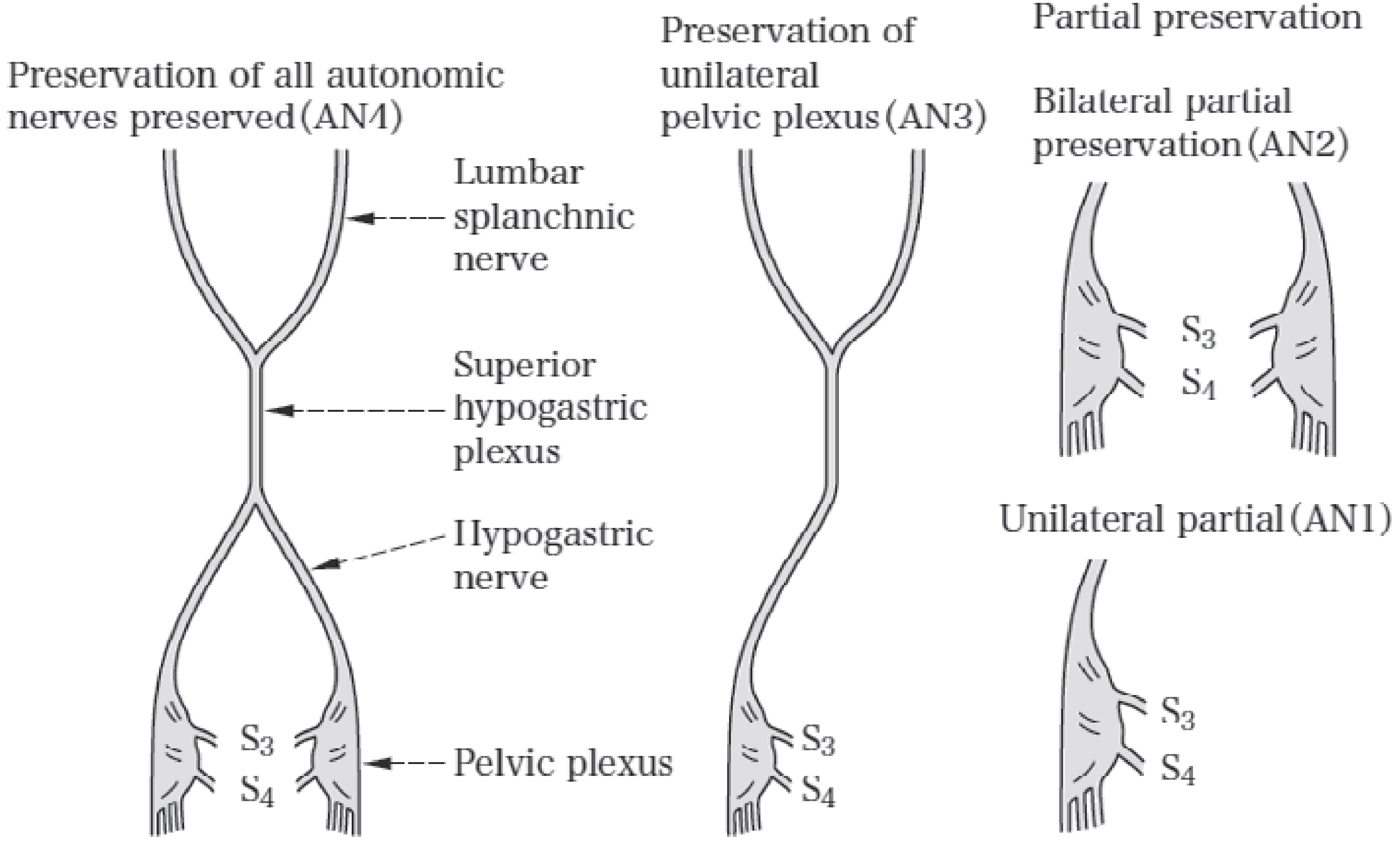
Preservation of all autonomic nerves preserved (AN4)
Lumbar splanchnic nerve
Superior hypogastric plexus
Hypogastric nerve
Pelvic plexus
S_3
S_4
Preservation of unilateral pelvic plexus (AN3)
S_3
S_4
Partial preservation
Bilateral partial preservation (AN2)
S_3
S_4
Unilateral partial (AN1)
S_3
S_4
