# Supplementary figures and images for: Effect of autonomic nervous system resection extent on urinary dysfunction in robotic rectal cancer surgery
Source: Ann Gastroenterol Surg. 2024 Nov 4;9(3):476–85. doi: 10.1002/ags3.12878 (PMC12080188; doi:10.1002/ags3.12878)

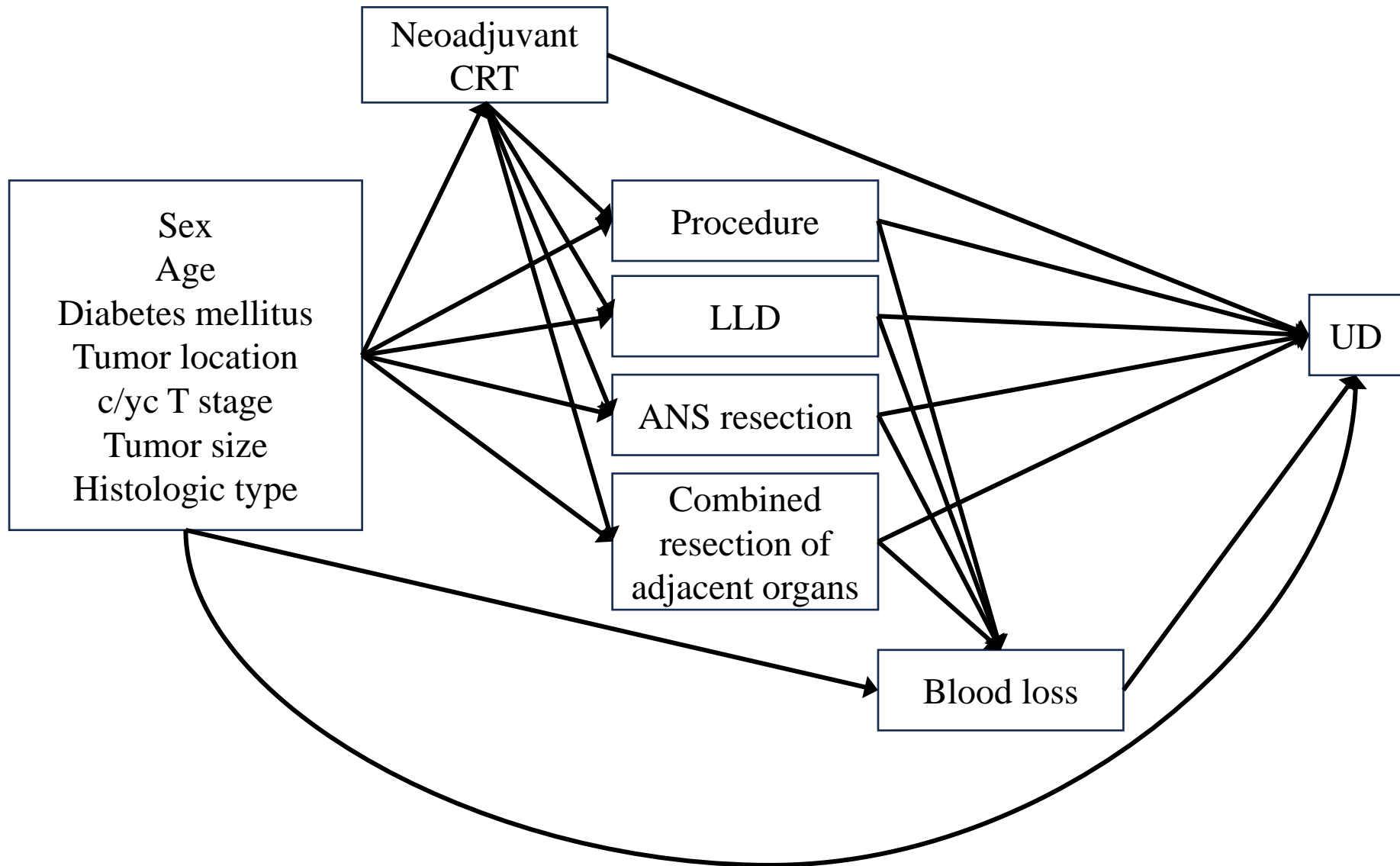

Supplement: Supplementary file 2 — Figure S2: Directed Acyclic Graph (DAG) on UD. [file AGS3-9-476-s002.pdf]

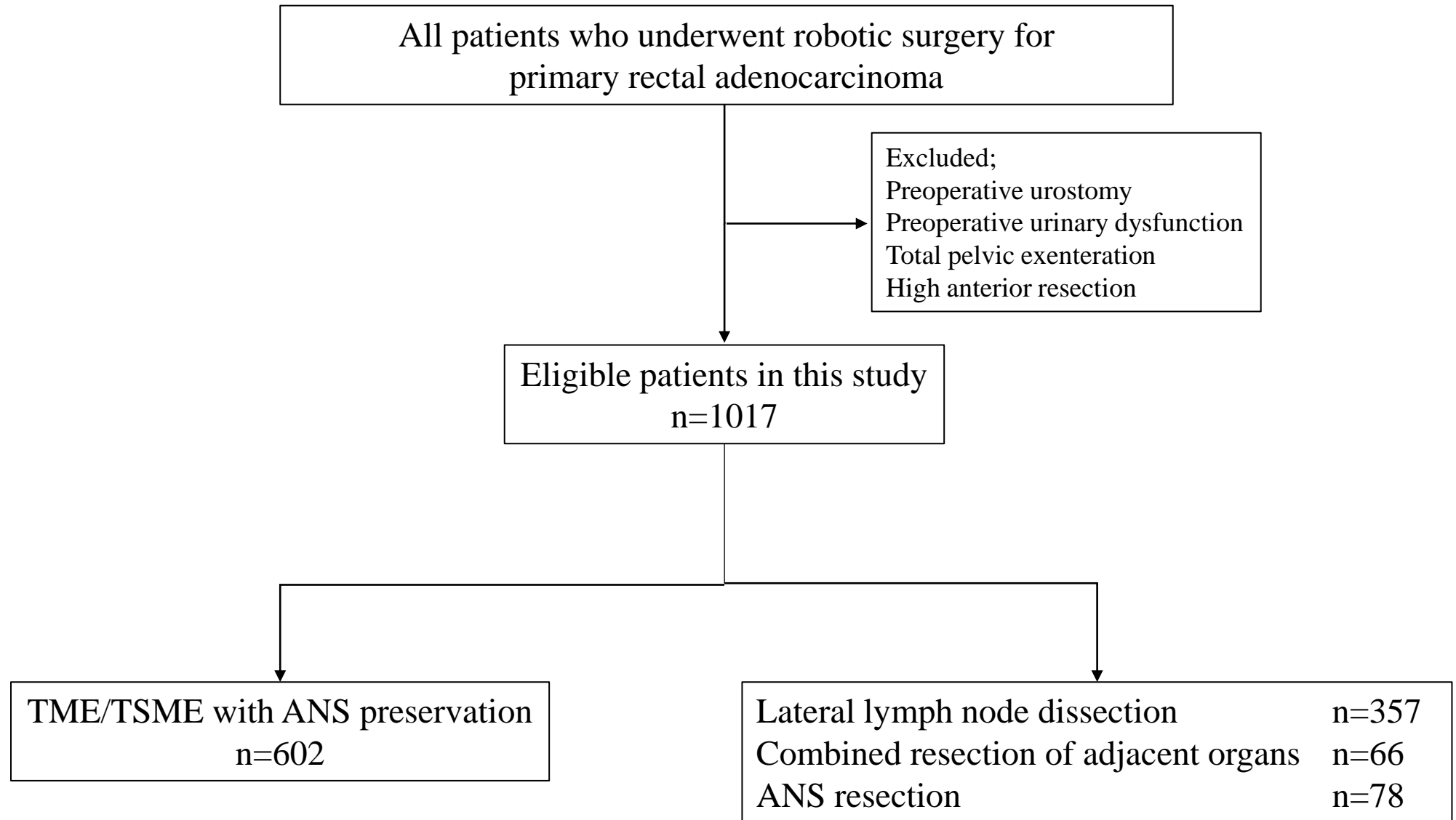

\*Included overlap

Supplement: Supplementary file 3 — Figure S3: Patient flow chart. [file AGS3-9-476-s003.pdf]
